# A New Controlled Release System for Propolis Polyphenols and Its Biochemical Activity for Skin Applications

**DOI:** 10.3390/plants10020420

**Published:** 2021-02-23

**Authors:** Eleni Spanidi, Athanasios Karapetsas, Georgia-Persephoni Voulgaridou, Sophia Letsiou, Nektarios Aligiannis, Ilias Tsochantaridis, Spyridon Kynigopoulos, Maria Lambropoulou, Ioannis Mourtzinos, Aglaia Pappa, Konstantinos Gardikis

**Affiliations:** 1 Research and Development Department, APIVITA SA, Industrial Park Markopoulo Mesogaias, 19003 Athens, Greece; spanidi-e@apivita.com (E.S.); letsiou-s@apivita.com (S.L.); 2 Department of Molecular Biology & Genetics, Democritus University of Thrace, 68100 Alexandroupolis, Greece; karapetsas_than@yahoo.gr (A.K.); georgiavou_85@hotmail.com (G.-P.V.); iliatsoc@gmail.com (I.T.); apappa@mbg.duth.gr (A.P.); 3 Department of Pharmacognosy and Natural Products Chemistry, Faculty of Pharmacy, National and Kapodistrian University of Athens, Panepistimiopolis Zografou, 11527 Athens, Greece; aligiannis@pharm.uoa.gr; 4 Laboratory of Histology and Embryology, Faculty of Health Sciences, School of Medicine, Democritus University of Thrace, 68100 Alexandroupolis, Greece; spyroskinigopoulos@hotmail.com (S.K.); mlambro@med.duth.gr (M.L.); 5 Department of Food Science and Technology, Faculty of Agriculture, Aristotle University of Thessaloniki, 54124 Thessaloniki, Greece; mourtzinos@agro.auth.gr

**Keywords:** propolis, controlled release, liposome, polyphenols

## Abstract

Propolis is a resinous substance produced by bees that exhibits antimicrobial, immunostimulatory and antioxidant activity. Its use is common in functional foods, cosmetics and traditional medicine despite the fact that it demonstrates low extraction yields and inconsistency in non-toxic solvents. In this work, a new encapsulation and delivery system consisting of liposomes and cyclodextrins incorporating propolis polyphenols has been developed and characterized. The antioxidant, antimutagenic and antiaging properties of the system under normal and UVB-induced oxidative stress conditions were investigated in cultured skin cells and/or reconstituted skin model. Furthermore, the transcript accumulation for an array of genes involved in many skin-related processes was studied. The system exhibits significant polyphenol encapsulation efficiency, physicochemical stability as well as controlled release rate in appropriate conditions. The delivery system can retain the anti-mutagenic, anti-oxidative and anti-ageing effects of propolis polyphenols to levels similar and comparable to those of propolis methanolic extracts, making the system ideal for applications where non-toxic solvents are required and controlled release of the polyphenol content is desired.

## 1. Introduction

Nowadays, there is a huge interest on natural products that can promote the state of health and well-being for humans. Bee products represent a significant category of natural products that exhibit specific health benefits. All bee products—honey, royal jelly, propolis, bee pollen, beeswax and even bee venom—have been largely investigated for their healing properties [1,2,3,4].

Propolis is a resinous substance which is produced by bees and presents many challenges in respect to its extraction and formulation process. It exhibits antimicrobial, immunostimulatory and antioxidant activity and is employed in the preparation of functional foods and cosmetics as well as in traditional medicine [2,5,6,7]. Its composition varies depending on the flora of the foraging region of the bees and the collection season. It contains approximately 50% resins, 30% waxes, 10% aromatic components, 5% pollen and 5% various other components [2,8,9]. The bioactive components of propolis are polyphenols, terpenes, steroids, as well as sugars and aminoacids [10,11,12,13,14,15]. Major polyphenols are flavonoids and phenolic acids. The complexity of the structure of propolis combined with the variable composition depending on the region and the collection season make both raw propolis and its extracts particularly difficult to formulate into products for per os or skin applications [6,16]. The extraction of propolis usually has a low yield in active component concentration [17,18], especially when natural solvents, as water or vegetable glycerol are used. The reason is mainly the hydrophobicity of the majority of propolis’ active ingredients.

In order to increase the extraction yield, various extraction methods have been developed [19,20] as well as methods of encapsulating its components in various carriers [21,22].

As far as the encapsulation of propolis is concerned, Zhang et al. [23] developed propolis loaded zein/caseinate/alginate nanoparticles that demonstrated a promising clean and scalable strategy to encapsulate propolis for applications in foods, supplements and pharmaceuticals. Ong et al. [24] prepared a chitosan-propolis nanoformulation based on optimum physicochemical properties such as particle size, zeta potential, polydispersity index, encapsulation efficiency and the rate of release of the active ingredients for biofilm applications. Do Nascimento et al. [25] developed nanoparticles (200–800 nm) loaded with red propolis extract. This copolymeric matrix system was able to encapsulate different flavonoids from red propolis extract with interesting characteristics of solubility and antioxidant activity demonstrating activity against leishmaniasis.

Liposomes is another form of carrier that has been investigated for propolis encapsulation. Aytekin et al. [26], developed a propolis loaded liposomal system that showed interesting results as a topical application in wound treatment having antioxidant and antimicrobial effects. Tao et al. [27], investigated the immune modulatory function of propolis flavonoids encapsulated in liposomes and showed the potential of the application of the formulation as an adjuvant. Arafa et al. [28], prepared oromuco-adhesive films for buccal delivery of propolis entrapped in niosomes. The results demonstrated controlled and targeted delivery of active ingredients against oral ulcers.

Cyclodextrins have been also used in order to encapsulate propolis ingredients [29,30,31,32]. Vasilaki et al. [33], prepared extracts of propolis by using aqueous solution of hydroxypropyl-beta-cyclodextrins as an alternative for food preservation. Ishida et al. [34], complexed caffeic acid phenethyl ester from propolis with γ-cyclodextrin in order to increase the stability of the former that gets easily degraded by esterases. The complex demonstrated significant in vitro activity against a range of cancer cells. γ-cyclodextrin was also used by Rimbach et al. [35] for the encapsulation of Brazilian green propolis supercritical extract. The system demonstrated promising properties for hepatoprotection helping the combat of chronic inflammation.

Liposomes loaded with cyclodextrin-bioactive molecule complexes are widely used as drug delivery systems, enhancing low aqueous solubility and stability [36,37]. Though such system has been never used for the encapsulation of propolis.

In the present study, an extraction and encapsulation of components of propolis in a combinatorial liposome–cyclodextrin system is performed. The particular properties of both carriers that were taken in advantage are: The ability of the cyclodextrins to enclose polyphenols and enhance their permeability through the skin [38,39] as well as the ability of the liposomes to encapsulate large quantities of components of various degrees of polarities, for topical transport of components and control of their release rate [40,41,42].

Based on our previous studies we have identified promising propolis extracts collected from different areas in Greece. The extracts demonstrated significant in vitro antioxidant activity due to their high total phenolic and flavonoid content. A sample collected from the area of Olympus mountain exhibited significant protection against the cytotoxic effects of UVB radiation by remarkably reducing DNA and protein oxidation damage levels in human immortalized keratinocyte (HaCaT). It also demonstrated significant antiaging efficacy as it decreased histological damage and lowered the induced expression of certain metalloproteinases (MMPs) following exposure to UVB in a reconstituted skin model [43].

The aim of the present study is to evaluate the in vitro effects of a controlled release delivery system of propolis polyphenols (CRPP) containing active propolis fractions previously characterized for their beneficial properties and to investigate the potential applications of such a system in skin applications. For this purpose, the potential antioxidant, antimutagenic and antiaging properties of the CRPP under normal and UVB-induced oxidative stress conditions were investigated in cultured skin cells and/or reconstituted skin model in order to analyze the biological activities of the CRPP of dermatological interests. In addition, to gain an insight into the molecular mechanism of activity of the CRPP, we studied transcript accumulation for an array of genes involved in many skin-related processes.

## 2. Materials and Methods

### 2.1. Materials

The system of extraction solvents consisted of ultrapure water (produced at APIVITA with a Reverse Osmosis System), 1.3 propanediol supplied by Connect Chemicals, Vimercate (MB), Italy, hydroxypropyl beta cyclodextrin from Gangwal Chemicals Pvt. Ltd., Maharastra, India, liposome, Pro-LipoTM Neo (propanediol 75.0% w/w, lecithin 25.0% w/w, tocopherol 0.25% w/w, Helianthus Annuus (sunflower) seed oil 0.15%w/w) was provided by Generex Pharmacist Pvt. Ltd. Mumbai, India/Lucas Meyer Cosmetics, Champlan, France, Hydrolite^®^ 5 Green (Pentylene Glycol) was provided by Symrise, Holzminden, Germany, Trilon^®^ BD (Disodium EDTA) supplied by BASF Hellas S.A., Thessaloniki, Greece. Organic certified raw propolis from Greek cultivation (origin: Olympus mountain) was used. For the maximization of the encapsulation efficiency, raw propolis must contain total polyphenol compounds more than 16 μg GA/mL after dissolution of 5% (v/w) propolis in methanol (PanReac, AppliChem, Darmstadt, Germany) and subsequent measurement in a spectrophotometer by the Folin–Ciocalteu method.

#### 2.1.1. Formulation of Controlled Release Propolis Polyphenols—CRPP System

In order to prepare the CRPP system, propolis was initially micronized (<1 mm) after being frozen for 24 h.

The quality specification of the ultrapure water was ≤1 μS/cm at 25 °C, which meets the specification of European Pharmacopoeia for the preparation of parenteric pharmaceutical products. This quality was necessary for full absence of loads in the final formulation which would otherwise cause the lipid membranes of the liposomes to aggregate and finally lead to a decrease in the product stability.

Thereafter, the micronized propolis was dispersed at a rate of 1 kg/min in the solvent system under stirring at 500–1500 rpm with industrial mixers (IKA^®^-Werke GmbH & Co. KG, Staufen im Breisgau, Germany). The propolis concentration was 10% (w/w). The system of extraction solvents consisted of deionized water and 1.3-propanediol at a ratio of 1.3 propanediol/water: 45/55. In the deionized water, hydroxypropyl-β-cyclodextrin had been predissolved at 5% w/w.

After a week the propolis was removed and the extract is filtered through a 25 μm nylon bag filter. As the system of propolis/cyclodextrin/solvents was under intense stirring at 3000 rpm (Homogenizer, Silverson, Chesham, England) at room temperature, the liposomal suspension was added thereto at a proportion of 3.0% (w/w). If required, pH adjustment in the range of pH 5–8 was previously performed, depending on the propolis used. The liposomal suspension added consisted of large unilamellar liposomes, lipidic bilayers and natural 1.3-propanediol.

The extract was stored at 6 °C for 3 days and, thereafter, filtered through a 0.22 μm millipore membrane filter (MF-Millipore™ Membrane Filter, 0.22 µm pore size, Merck KGaA, Darmstadt, Germany). Unentrapped propolis was removed by gel permeation chromatography (Sephadex G75, Sigma-Aldrich, MI, USA) and the system was stored in a dark-colored container at temperature of 6 °C.

#### 2.1.2. Physicochemical Characterization of CRPP

The CRPP system was characterized as described by Gardikis et al. (2010) [44]. The hydrodynamic diameter of the CRPP system was measured by light scattering. Then, 100 μL of the liposomal suspension was 30-fold diluted immediately after preparation or after reconstitution and z-average mean and ζ-potential of the formulations were measured. Samples were scattered (633 nm) at 90° angle, and measurements were made at 25 °C in a photon correlation spectrometer (Zetasizer 3000HS, Malvern Instruments, Malvern, UK) and analyzed by the CONTIN method (MALVERN software).

#### 2.1.3. Propolis Encapsulation Efficiency in CRPP

The encapsulation efficiency in delivery systems usually refers to the amount of drug i.e., pure bioactive compound encapsulated in the system [45].

In our case, as the term “drug” is of no use we used total polyphenol count as a marker, modifying the encapsulation efficiency percentage to:

EE = entrapped polyphenols/initial polypenols × 100 (1)

Εntrapped polyphenols is the total phenolic content measured in the final system, as described in Section 2.1.4. Initial poyphenols refers to the total phenolic content of the raw propolis when dissolved in methanol, as described by Karapetsas et al. [43].

#### 2.1.4. Total Phenolic Content (TPC)

The phenolic content of the CRPP samples, was assessed by employing the Folin–Ciocalteu (F-C) method. The Folin–Ciocalteu solution was prepared with 10% dilution in distilled water and the alkaline environment was achieved with the addition of 7.5% sodium carbonate in purified water. In a 96 well plate, 25 μL of sample, 125 μL Folin–Ciocalteu solution and 100 μL Na_2_CO_3_ solution were mixed. The plates were incubated for 30 min at ambient temperature in the dark. Absorbance was measured at 765 nm, using TECAN microplate reader. Total phenolic content was estimated from a standard curve of absorbance values derived from standard concentration solutions of gallic acid (GA). Twelve serial dilutions (1.25, 2.5, 5, 10, 20, 30, 40, 50, 60, 70, 80 and 100 μg/mL final concentrations) were prepared from a stock solution of 10 mg/mL in DMSO. Samples were initially tested at 4 different dilutions (initial sample/water; 1:0, 1:5, 1:10 and 1:15) in order to exhibit absorbance value in the linear part of the curve. Total content of phenolic compounds in the tested samples was determined from the linear regression equation of standard curve (y = 0.0629 x + 0.0518, R^2^ = 0.989) and expressed in gallic acid equivalents (μg GA/mL). The experiments were performed in triplicates.

#### 2.1.5. Antioxidant Activity by 2,2-diphenylpicrylhydrazyl (DPPH)

The antioxidant activity of the CRPP samples, was determined using DPPH as a free radical. CRPP extracts (0.1 mL) was added to 3.9 mL of a 6 × 10^−5^ mol/L DPPH methanol solution. The decrease in absorbance was determined at 515 nm. The antioxidant activity was estimated from a standard curve of absorbance values derived from standard concentration solutions of Trolox.

Trolox Equivalents (mM) = 0.0177% DA_515nm_ + 0.08 R^2^ = 0.9998 (2)

where

% DA_515nm_ = Abs_515nm(t=0)_ – Abs_515nm(t=30)_/Abs_515nm(t=0)_ × 100 (3)

#### 2.1.6. In Vitro Release Studies of Propolis Polyphenols from CRPP

Τhe release rate of the polyphenols of propolis was measured in a buffer solution at pH 7.2 at 37 °C.

An in vitro study of the release of the polyphenols of propolis was performed by using dialysis sacks.

Then, 1 mL of the CRPP extract was placed in the dialysis sacks of a MWCO = 1000 (Pur-A-Lyzer, Midi Dialysis Kit, Sigma-Aldrich, Missouri, USA). The sack was placed in buffer pH = 7.2 (CARLO ERBA Reagents, Val-de-Reuil, France) which was constantly stirred with a small magnetic bar and thermostated at 37 °C throughout the experiments (Hot plate magnetic stirrer, IKA^®^ Werke GmbH & Co, Staufen im Breisgau, Germany). On specific time points (15′, 30′, 45′ and 1, 2, 4, 8, 25, 48 h), samples were taken, and their polyphenol concentration and antioxidant activity were measured. The buffer quantity removed is replaced by buffer pH = 7.2 at temperature of 37 °C to maintain the sink conditions.

#### 2.1.7. Stability of CRPP

Physical stability test of the CRPP was carried out for six months at various conditions at room temperature (RT), 6 °C and 38 °C. The parameters that were determined were pH (Seven Compact, Mettler Toledo, OH, USA), refractive index (% Brix) (RX-5000α, ATAGO CO., LTD., Tokyo, Japan), density (DMA 38, Anton Paar GmbH, Graz, Austria) and organoleptic characteristics like aspect, color and odor. Physical parameters were examined by visual examination comparing with the initial sample.

The parameters that were determined in order to investigate the chemical stability was antioxidant activity by DPPH (Sigma-Aldrich, MI, USA) scavenging assay [46], and total phenolic compounds by the Folin–Ciocalteu method (Merck KGaA, Darmstadt, Germany) [47] at appropriate time intervals (4, 8 and 24 weeks).

### 2.2. Cell Lines and Culture

The spontaneously immortalized human keratinocyte cell line HaCat (ATCC, Rockville, MD, USA) was maintained in Dulbecco’s Modified Eagle’s Medium high glucose, supplemented with 10% fetal bovine serum (FBS), 100 μg/mL streptomycin and 100 U/mL penicillin (all purchased from Biosera, Boussens, France). Cells were cultivated with 5% CO_2_ at 37 °C in a humidified incubator. Moreover, primary human dermal fibroblasts (NHDF) were used. NHDF cells were isolated from normal human adult skin and purchased from Lonza Clonetics^TM^ (Lonza, Walkersville, MD, USA) [48]. The cells were grown in a recommended media FGM™-2 BulletKit™ that contain 2% serum according to Lonza instructions. Cells were grown for 2 days and subculturing happened when the cells were 70–80% confluent.

### 2.3. Cytotoxicity Assessments

#### 2.3.1. Sulforhodamine B (SRB) Assay

SRB assay was performed as described earlier [43]. Briefly, 5 × 10^3^ HaCat cells per well were seeded in 96-well microplates 24 h prior to the experiment and then treated with a range of CRPP concentrations (0–5% v/v), prepared in culture medium. Following a 72 h incubation period, cells were fixed with 50% (w/v) trichloroacetic acid (TCA) (Applichem, Darmstadt, Germany) at 4 °C for 1 h and stained with 0.4% (w/v) SRB (Sigma-Aldrich, Dorset, UK) diluted in 1% (v/v) acetic acid (Scharlau, Barcelona, Spain) for 30 min. The excess dye was removed via washing with 1% (v/v) acetic acid. Plates were then left to dry (overnight) and the dye was dissolved in 10 mM Tris base solution (Sigma-Aldrich, Dorset, UK). Optical density was determined at 570 nm using a microplate reader (Tecan, Männedorf, Switzerland). The percent cellular survival was calculated using the formula:

[(sample OD_570_ − media blank OD_570_)/(mean control OD_570_ − media blank OD_570_)] × 100 (4)

Sigma Plot Software (version 10) was used for estimating the EC_50_ value (the CRPP concentration where a 50% decrease in the control’s cell viability is observed) from the dose-response curves through the regression analysis via the four-parameter logistic curve. The EC10 value (the CRPP concentration where a 10% decrease in the control’s cell viability is observed) was calculated through the QuickCalcs online calculator (http://www.graphpad.com/quickcalcs/Ecanything1.cfm) by GraphPad Software with the formula:

EC_F_ = (F/(100 − F))^√H × EC_50_ (5)

where ECF is the EC value of interest and H the hill slope value of the dose response curve.

#### 2.3.2. Measurement of Intracellular ATP Levels

We assessed the effect of CRPP on the viability of fibroblasts based on intracellular ATP determination. ATP was measured using the ViaLight HS BioAssay kit (Lonza, Walkersville, MD, USA) according to the manufacturer’s protocol. Briefly, 3.5 × 10^3^ NHDF cells per well were seeded in 96-well microplates 24 h prior to the experiment and then treated with different CRPP concentrations (0–3% v/v), prepared in culture medium. After a 48 h incubation, ATP intracellular levels were determined in a GloMax 20/20 single-tube luminometer (Promega, Madison, WI, USA) for 1 s.

### 2.4. UVB Assessments

#### 2.4.1. Single Cell Gel Electrophoresis Assay (Comet Assay)

Comet assay was conducted as described previously [43] with few modifications. Specifically, 3 × 105 HaCat cells were seeded and cultured on 60 mm plates 24 h prior to the experiment. Cells were then washed with Phosphated buffer saline (PBS) (w/o Ca^2+^/Mg^2+^) (Biosera) and irradiated with 55 mJ/cm^2^ UVB (12 s) with a Bio-Link BLX254 Crosslinker (in PBS) (Vilber Lourmat, Marne-la-Vallée, France). Cells were allowed to recover for 16 h either with culture medium alone or with the culture medium supplemented with 0.1% v/v CRPP. As a control, a group of non-irradiated cells was also included in the experiment.

Subsequently cells were collected through trypsinization, washed and diluted in ice-cold PBS (2 × 104 cells/mL of PBS w/o Ca^2+^/Mg^2+^) and 0.4 mL of the diluted cells were then suspended in 1.2 mL low-gelling-temperature agarose (1% (w/v) in ddH_2_O) and coated to super frosted glass microscope slides precoated with a layer of 1% low-melting point agarose (warmed to 37 °C prior to use) and allowed to set for at least 2 min at RT. Cells were then lysed in lysis solution (1.2 M NaCl, 100 mM Na_2_EDTA, 0.1% sodium lauryl sarcosinate and 0.26 M NaOH, pH = 13) for 1 h at 4 °C in the dark to achieve the denaturation of the two DNA strands (alkaline unwinding). Following lysis, slides were washed twice with rinse solution (0.03 M NaOH and 2 mM Na_2_EDTA, pH~12.3) for 20 min at RT, subjected to electrophoresis for 25 min (in the presence of rinse solution) at 0.6 V per cm between the positive and negative electrodes of the electrophoresis apparatus. Slides, were subsequently neutralized in ddH_2_O, stained with propidium iodide (10 μg/mL in ddH_2_O) for 20 min and processed for observation on a Nikon ECLIPSE E200 fluorescence microscope.

For the estimation of DNA damage 100 comets of each slide were scored as class 0 to 4 in proportion to the observed tail due to DNA damage, observed “tail” as described previously (Collins A. et al. 1997). Each comet was assigned a value on the basis of its class and the overall score of 100 comets ranged from 400 (100% comets of class 4) to 0 (100% comets being in class 0). In this way, the overall DNA damage of the sample was expressed in arbitrary units.

#### 2.4.2. Assessment of Protein Carbonyl Content

Protein carbonyl colorimetric assay kit (Cayman Chemical) was used for estimating protein oxidation through the detection of hydrazone (at 370 nm) as a result of the 2,4-dinitrophenylhydrazine (DNPH) reaction with the protein-bound carbonyl groups.

Specifically, 2.5 × 106 HaCat cells were seeded in 100 mm plates and cultured for 24 h before they were washed with PBS (Biosera, Boussens, France) and irradiated with 55 mJ/cm^2^ UVB. Cells were subsequently treated either with culture medium alone or with culture medium supplemented with 0.1% v/v of CRPP for 24 h, while a group of non-irradiated cells was also used in the experiment as a control group. Cells were then collected and lysed through sonication in ice-cold lysis buffer (50 mM MES, pH 6.7 and 1 mM Ethylenediaminetetraacetic acid (EDTA)). Cell lysates were then centrifuged at 10,000× *g* for 15 min (4 °C) and 200 μL of the supernatants were used with 800 μL DNPH for test samples or with 800 μL 2.5 M HCl for control samples. All samples were incubated in the dark (at RT) for 1 h, fixed through the addition of 1 mL 20% TCA and incubated for 5 min at 4 °C. Samples were then centrifuged at 10,000× *g* for 10 min at 4 °C and pellets were washed once with 10% TCA and subsequently three times with the ethanol/ethyl acetate mixture. Protein pellets were then re-suspended in guanidine hydrochloride and the absorbance at 370 nm was finally measured with an Enspire Multimode plate reader (PerkinElmer, Inc., Seer Green, UK). The protein carbonyls concentration was estimated through the formula:

Conc.(nmol/mL) = [(CA)/(0.011 mM^(−1))] × (500 μL/200 μL)(6)

where CA is the absorbance of the test sample after subtracting the absorbance of the control.

NHDF cells were subjected to UVB irradiation (UVB = 312 nm). Specifically, 3.5 × 103 NHDF cells were seeded in 6-well plates and cultured for 24 h in culture medium before a 48 h incubation with 1% v/v CRPP. After incubation and prior to UVB exposure the cells were washed two times with PBS (Life Technologies, Thermo Fisher Scientific, Waltham, MA, USA) to prevent direct intramolecular reactions. Then PBS was added to the cells and the cells were exposed for 20 min, at UVB irradiation. During UVB treatment a control was included. After the UVB exposure culture media was added for an additional 20 min, finally, the ATP levels were assessed following the previously mentioned procedure. The radiation dose of 0.3 J/cm^2^ for UVB irradiation. UV irradiation was achieved was a UV-lamp VL-8.LM (Vilber Lourmat, Collégien, France) in two wavelengths.

### 2.5. Quantitative Real Time PCR(QPCR)

QPCR was performed as described earlier [43]. Total RNA was extracted either from skin tissues or from HaCat cells using Trizol reagent (Life Technologies, Thermo Fisher Scientific, Waltham, MA, USA) according to manufacturer’s instructions and the quantity and quality of the isolated RNA was evaluated both spectrophotometrically and through agarose gel electrophoresis. For cDNA synthesis, approximately 5 μg of total RNA was reverse-transcribed into cDNA with Superscript First-Strand Synthesis Kit (Life Technologies). For real-time PCR analysis, the KAPA SYBR^®^ FAST qPCR Kit (Kapa Biosystems, Wilmington, DE, USA) was used according to the manufacturer’s instructions, while the reactions were performed on an Applied Biosystems Step One Instrument in MicroAmp^®^ Fast Optical 48-Well Reaction Plates (both from Applied Biosystems, Thermo Fisher Scientific) under the following conditions: 95 °C for 3 min, 95 °C for 15 s (for 40 cycles) and 60 °C for 1 min. Each reaction was performed in triplicates and each experiment included two non-template controls. The sequences of the primers are provided in Table 1. Primer specificity was verified by melting curve analysis. Expression data were normalized to beta-actin using the 2^−ΔΔCt^ method [49].

#### 2.5.1. RNA Isolation and cDNA Synthesis-NHDF Cells

Total RNA was isolated from primary normal human dermal fibroblasts using a kit by Norgen BIOTEK Corporation (Schmon Pkwy, ON, Canada). RNA according to manufacturer’s instructions. Then it was quantified using a NanoDrop at 260/280 nm and 260/230 nm. The RNA quality was confirmed on 1% agarose gel. cDNA was synthesized from 0.8 μg total RNA using reversed transcription according to Super-Script II (Invitrogen, Paisley, United Kingdom) protocol. The reaction mixture was incubated at 42 °C for 50 min, followed by heat inactivation at 70 °C for 15 min using oligo (dT) primers.

#### 2.5.2. Primer Design and RT-PCR Analysis

Target cDNAs were amplified using gene specific primers and designed from the transcribed region of each gene using Primer Express 1.5 software (Applied Biosystems, Darmstadt, Germany). Quantitative RT-PCR reactions were performed on the Stratagene MX3005P using iTaq™ fast SYBR^®^ Green supermix with ROX (BioRad Laboratories, Hercules, CA, USA), gene specific primers at a final concentration of 0.5 μΜ each and 1 μL of the cDNA as template. Detailed information for all the genes in this study, including names, gene symbols, accession numbers, predicted topology and gene specific primers, are presented in supplemental data set. PCR cycling started at 95 °C for 10 min, followed by 40 cycles of 95 °C for 15 s and 60 °C for 1 min. The primer specificity and the formation of primers-dimers were monitored by dissociation curve analysis and agarose gel electrophoresis on a 4% (w/v) gel. Actin beta (ACTB) and glyceraldehyde-3-phosphate dehydrogenase (GADPH) were used as genes internal standards. Relative transcript levels of the gene of interest (X) were calculated as a ratio to the internal standard gene transcripts (H), as (1 + E) ^– ΔCt^, where ΔCt was calculated as (CtX – CtH). PCR efficiency (E) for each amplicon was calculated employing the linear regression method on the log (fluorescence) per cycle number data, using LinRegPCR software [50]. All real-time quantitative PCRs (qPCRs) were performed on three biological repeats.

### 2.6. Human Reconstituted Skin Model (EpiDermTM EPI-200)

EpidermTM EPI-200 is a human, normal epidermal tissue 3D model. It consists of human-derived, normal epidermal keratinocytes that have been cultured to reconstitute a multilayered epidermal model. EpidermTM EPI-200 is both metabolically and mitotically active and it histologically mimics human skin. The EpidermTM EPI-200 was purchased by MatTek as 24-well culture plate inserts with each insert containing skin tissue. Upon receipt the skin tissues were equilibrated for 24 h at 37 °C in humidified atmosphere (5% CO_2_) in EPI-100 assay medium, in which they were further maintained. Throughout all the experiments skin models were cultured in 6-well plates at the air-liquid interface, with the lower surface exposed to the EPI-100 Assay medium and the apical surface exposed to air.

#### 2.6.1. Treatment and UVB Irradiation of EpiDermTM EPI-200

EpidermTM EPI-200 tissues were topically treated in the apical surface with 0.1% (v/v) CRPP (diluted in the EPI-100 Assay medium) for 2 h and then washed 3 times with 1 times PBS through gentle pipetting. Then, the culture media, in which the lower surface was exposed, was replaced with PBS, the skin tissues were irradiated with 55 mJ/cm^2^ UVB and they subsequently treated in the apical surface again with 0.1% (v/v) CRPP for 2 h. Finally, culture inserts were placed in fresh EPI-100 Assay medium and skin tissues were allowed to recover for 24 h.

#### 2.6.2. Immunohistochemistry (IHC)

Immunohistochemistry was performed as described earlier [43]. For immonohistochemistry EpidermTM EPI-200 tissues were collected 24 h post UVB irradiation and treatment with CRPP, fixed in formalin and then embedded in paraffin. Tissue sections of two-micron (2 µm) were then deparaffinized, rehydrated and treated with 0.3% H_2_O_2_ in methanol (5 min) to prevent endogenous peroxidase activity and were subsequently immunostained by the peroxidase method (Envision System, DAKO, Carpinteria, CA, USA) according to the manufacturer’s instructions. In brief, after antigen retrieval and endogenous peroxidase blockade, sections were blocked with Protein Block Serum-Free (DAKO) and incubated overnight (4 °C) with the monoclonal or polyclonal antibodies against MMP-1 (mouse-raised), MMP-3 (rabbit-raised), MMP-9 (mouse-raised) (all from Acris, Herford, Germany) and MMP-7 (Proteintech, Machester, UK) in 1:750, 1:100, 1:900 and 1:100 dilutions, respectively. Sections were then incubated with the respective secondary antibodies for 60 min (RT). Finally, sections were counterstained with 0.05% diaminobenzidine (10 min) and Mayer’s hematoxylin, mounted and examined under a Nikon Eclipse 50i microscope. Control slides were incubated for the same period with non-immunized rabbit or mouse serum. An independent pathologist evaluated the results on the basis of the percentage of stained cells in the sample.

#### 2.6.3. CRPP Incorporated in an Emulsion—Reconstructed Human Skin Model

We evaluated the potential skin irritation of cosmetic formulation based on CRPP with reconstructed human skin model. The cosmetic formulation was an oil in water basic cream base with 1% CRPP incorporated (Table 2). The parameters measured in vitro were the percentage cell viability using an MTT effective time 50 (ET_50_) assay after topical application of the products for different exposure times (2 h, 5 h and 18 h) according to manufacturer’s instructions. For negative control Dulbecco’s phosphate-buffered saline (DPBS) was used as well as for positive control 5% sodium dodecyl sulfate (SDS) was used. For negative control, 1.0% Triton X-100 was used.

### 2.7. Statistical Analysis

Graphs and statistical analysis of the data were performed with GraphPad Prism 5 and Sigma Plot Software v.10. Results are expressed as the mean ± SD of at least three independent experiments, which were performed, in triplicates, per sample for each condition tested. Statistical analysis between two individual groups were performed by two-tailed Student’s t-test. A *p* ≤ 0.05 was considered as statistically significant.

## 3. Results

### 3.1. Characterization of CRPP

The encapsulation efficiency of propolis polyphenols incorporation in the system was 84% ± 4% when the concentration of propolis in the system was 10% w/w. Any concentration above this point provided lower yields (data not shown) making the system insufficient carrier, at least for this specific propolis.

Table 3 depicts the mean hydrodynamic diameter, polydispersity index and ζ-potential of the system at day 1 and after 1 month at various temperatures. The values remain practically stable except the temperature of 38 °C where there is a slight but statistically significant increase in particle size.

As shown in Table 4, the initial total phenolic compounds (TPC) appear to decrease over time.

Values in week 4, show a statistically significant difference from the initial value, and the total phenolic compounds appears to decrease significantly at temperature 38 °C, although at the other two temperatures it does not appear to be affected. Τhe values of temperatures 6 °C and RT, do not appear to differ significantly from week 8 values. Although the total phenolic compounds decrease at RT, the difference does not appear to be significant. After 24 weeks, only the values at 6 °C temperature, do not seem to have decreased significantly. The perfect temperature to maintain the phenolic compounds seems to be the 6 °C.

As in TPC, so in the antioxidant activity, the initial value differs significantly from the rest of the values, which means that the antioxidant activity decreases significantly over time. At week 4, the different temperatures do not seem to affect the antioxidant activity of CRPP extract, as is the case with the values of the different temperatures for week 8. Furthermore, it seems that in the antioxidant activity, as well as in TPC, the temperature that presents the most stable values is that of 6 °C, as it does not decrease significantly from the 4th to the 8th week. Values in week 24 show a significant decrease compared to all other values.

### 3.2. In Vitro Release Studies

The cumulative release of the polyphenols at pH 7.2 and at a temperature of 37 °C was 23.99% ± 1.18% at 8 h.

**Figure 1:**
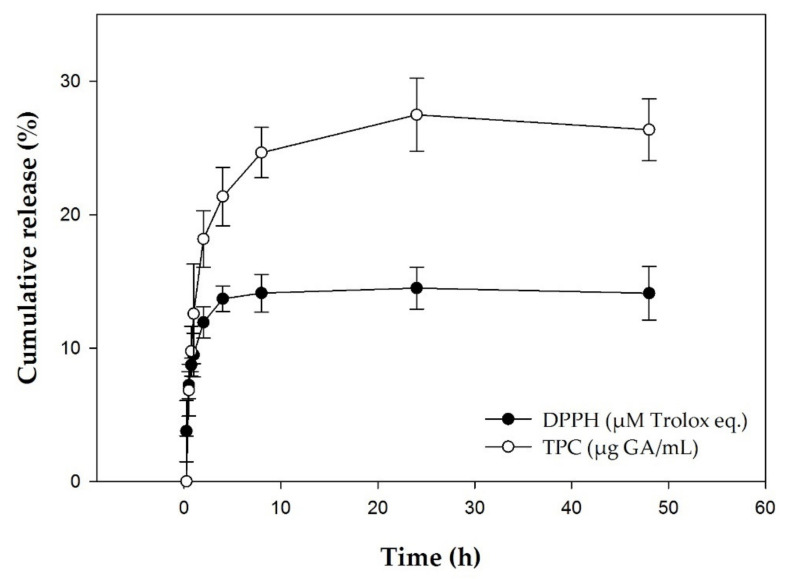
Control release of antioxidant activity (DPPH) and total phenolic compounds (TPC) of CRPP for time 0 to 48 h. The results are shown as the mean ± SD of three measurements.

In vitro release profile of CRPP extract inclusion system as a function is presented in Figure 1. The release of the total phenolic compounds and the antioxidant activity reach practically a plateau in 8 h.

### 3.3. Physical Chemistry

The results of the assessment of the organoleptic characteristics as well as pH value and refractive index of the CRPP extract are depicted in Table 5.

The parameters examined were found to be stable at different temperatures i.e., room temperature, 6 °C and 38 °C within 24 weeks. As acceptance criteria, formulations with pH value and refractive index variations higher than 15%, comparing with the initial value.

### 3.4. Cytotoxicity Profile of CRPP

**Figure 2:**
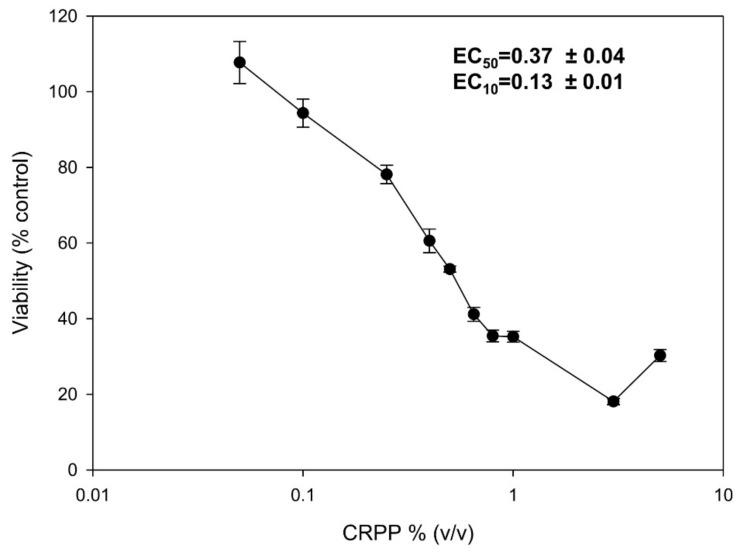
Cytotoxicity profile of CRPP on human immortalized keratinocyte (HaCat) cells. HaCat cells were incubated for 72 h with increasing concentrations (0%–5%) of CRPP and their cell viability was estimated via sulforhodamine B (SRB) assay. The EC_50_ and EC_10_ values (efficient concentration that causes 50% and 10% decrease in cell viability, respectively) of CRPP were determined from the dose-response curves. The results are shown as the mean ± SD of three independent experiments.

To determine the cytotoxicity profile of CRPP, we studied its effect on the viability of the human keratinocyte cell line HaCat as well as of NHDF. HaCat cells were incubated for 72 h with increasing concentrations of CRPP and SRB-base cell viability curves were plotted, which were used for calculating EC_50_ and EC_10_ values (Figure 2). Our data demonstrated that CRPP did not exhibited significant cytotoxicity against HaCat cells, while EC_50_ and EC_10_ values were 0.37 and 0.13% respectively. On the basis of these results a concentration of 0.1% (v/v) of CRPP was used for all subsequent experiments.

**Figure 3:**
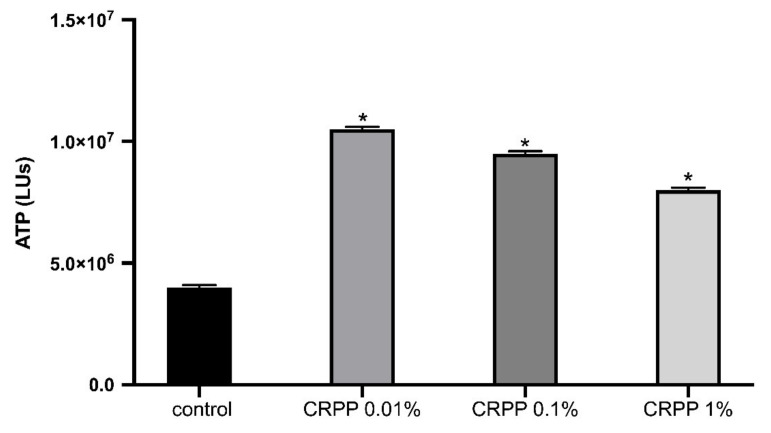
Intracellular levels of ATP (LUs). NDHF cells were incubated for 48 h with three different concentrations (0.01%, 0.1% and 1%) of CRPP. The results are shown as the mean ± SD of three independent experiments. *—*p* ≤ 0.05, significantly different from untreated cells.

Moreover, we assessed the effect of CRPP on the viability of NHDF cells based on intracellular ATP determination. NHDF cells were incubated for 48 h with three different concentrations of CRPP (0.01%, 0.1% and 1%) have shown an increase to the intracellular levels of ATP up to 100% compared to untreated cells (control) Figure 3).

### 3.5. CRPP Protects HaCat as Well as NHDF Cells under UVB Exposure Conditions

**Figure 4:**
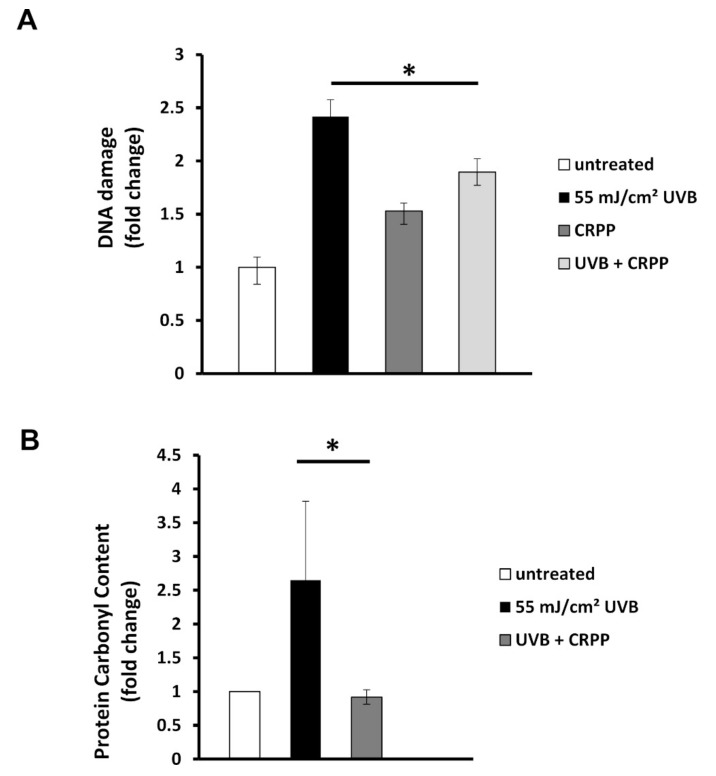
CRPP protects HaCat cells from UVB-induced DNA and protein oxidative damage. (**A**) HaCat cells were UVB-irradiated for 12 s (55 mJ/cm^2^) or left untreated. Irradiated and non-irradiated cells were incubated for 16 h either with 0.1% CRPP or with normal culture medium alone, and then subjected to single cell gel electrophoresis assay (Comet assay). The scored arbitrary units (AU) represent the extent of DNA damage in the untreated, the UVB-irradiated, the CRPP-treated and the UVB-irradiated and CRPP-treated cells. (**B**) protein oxidative damage was estimated by measuring the protein carbonyl levels of the untreated, the UVB-irradiated and the UVB-irradiated and CRPP-treated cells with a 2,4-dinitrophenylhydrazine (DNPH) colorimetric assay. The concentration of the protein carbonyls was determined, adjusted to the total protein concentration and expressed as fold change compared to the untreated cells. The data presented are the mean ± SD of three independent experiments performed in duplicates. * *p* ≤ 0.05, significantly different from the UVB-irradiated cells.

Then, we examine the extent of the UVB-induced DNA damage in HaCat cells, as well as their oxidative status, in the presence or absence of CRPP. Therefore, HaCat cells were irradiated with 55 mJ/cm^2^ UVB and then allowed to recover for 24 h in culture medium alone or supplemented with 0.1% (v/v) CRPP. DNA damage was assessed by single cell gel electrophoresis assay (comet assay), performed under alkaline conditions in order to detect both single and double DNA strand breaks. Our data demonstrated that UVB radiation exhibited a strong genotoxic effect on HaCat cells by increasing DNA damage levels approximately 2.5 times. Interestingly, treatment of the irradiated cells with CRPP resulted in a significant inhibition of the UVB-induced DNA damage, with the protective effect of CRPP to be approximately 22%. Finally, non-irradiated HaCat cells treated with CRPP exhibited only a slight increase in their DNA damage levels in comparison with the control (non-irradiated/non-treated HaCat cells) (Figure 4A).

The total protein carbonyl content was subsequently analyzed for estimating HaCat cells oxidative status. Protein carbonyls are a group of irreversible protein modifications formed during the early stages of the oxidation process, widely used as biomarkers of oxidative stress [51,52] Our data indicated that UVB exposure induced a significant increase in protein oxidation levels in HaCat cells, which was strongly inhibited as a result of CRPP treatment. In fact, CRPP restored total protein carbonyl content to the levels of the control-non irradiated HaCat cells (Figure 4B).

**Figure 5:**
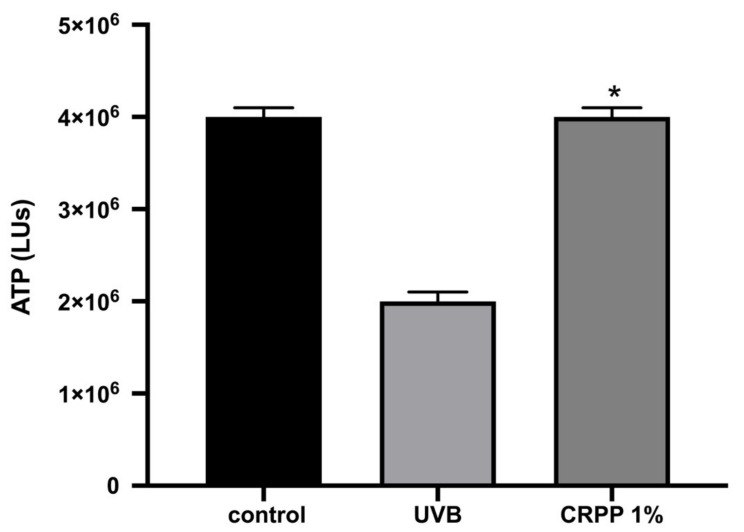
Intracellular levels of ATP (LUs) under UVB irradiation. Primary human dermal fibroblasts (NHDF) cells were UVB-irradiated for 20 min, at UVB irradiation (0.3 J/cm^2^) or left untreated. Prior to irritation cells were incubated for 48 h either with 1% CRPP or with normal culture medium alone, and then subjected to ATP assay to assess cell viability. The data presented are the mean ± SD of three independent experiments performed in duplicates. *—*p* ≤ 0.05, significantly different from the UVB-irradiated cells.

Furthermore, we evaluated the intracellular levels of ATP in NHDF cells under UVB treatment. According to Figure 5, the addition of 1% CRPP in NHDF cells has a protective effect on the cells under UVB treatment, since the intracellular levels of ATP are higher in control (untreated cells) and in NHDF under stress (NHDF cells treated with UVB).

CRPP affects the expression of genes related to cell proliferation, aging, immune response and inflammation. 

**Figure 6:**
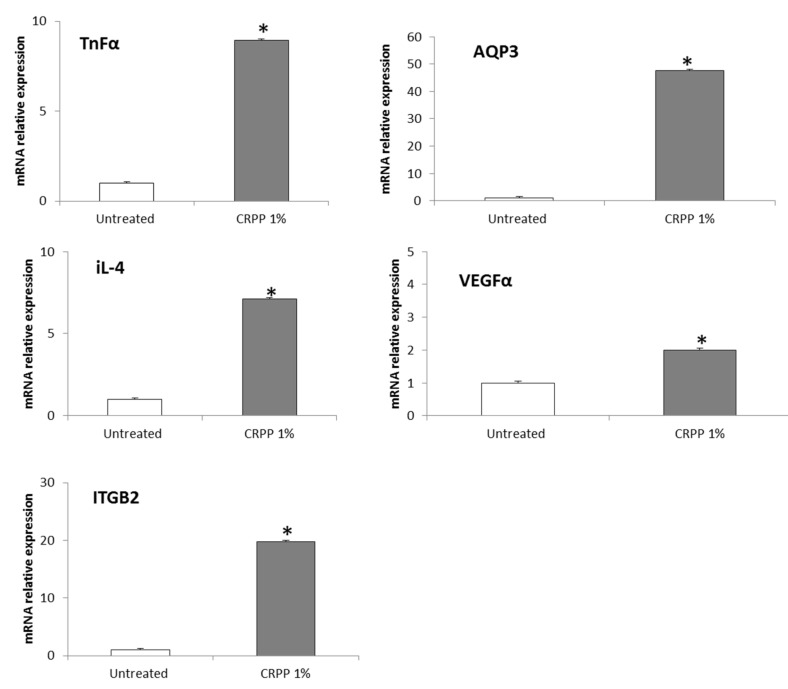
Expression analysis of candidate genes in the presence and absence of 1% v/v CRPP in NHDF cells. Transcript levels of genes encoding proteins, tumor necrosis factor (TNFα) human aquaporin-3 (AQP3), interleukin-4 (Il-4), vascular endothelial growth factor A (VEGFα) and integrin subunit beta 2 (ITGB2) are showed. Data corresponds to the mean ± SEM of at least three independent experiments. Gene expression levels were normalized in relation to intact cells and using the geometric mean of two reference genes (βactin and glyceraldehyde-3-phosphate dehydrogenase (GADPH)). * denote significantly different from untreated NHDF cells (control) of *p* < 0.05 (two tailed unpaired student t-test), respectively.

To understand, the molecular mechanisms associated with CRPP bioactivity, an RT-qPCR platform for an array of key-genes was deployed. Genes involved (number of total genes = 20) in primary cellular processes in human skin, such as cell proliferation, anti-oxidant and immune response, aging, inflammation and extracellular matrix (ECM) generation were assessed. Here we present, specific gene transcript levels showed significant changes under the experimental conditions (Figure 6). According to Figure 6, transcript levels of genes encoding proteins, tumor necrosis factor (TNFα) human aquaporin-3 (AQP3), interleukin-4 (Il-4), vascular endothelial growth factor A (VEGFα) and integrin subunit beta 2 (ITGB2) were upregulated with the addition of CRPP in NHDF cells compared to untreated NHDF cells (control) (*p* < 0.05).

### 3.6. CRPP Protects Human Reconstituted Skin Model from UVB-Induced Histological Lesions and Matrix Metalloproteinases Overexpression

Our previous results indicated the anti-oxidant and anti-mutagenic potential of CRPP in vitro. In order to also investigate the anti-ageing properties of CRPP, we utilized an ex vivo reconstituted human epidermal 3D model and examined the potential effect of CRPP treatment on the UVB-induced overexpression of several matrix metalloproteinases. EpidermTM EPI-200 consists of neonatal-derived, normal epidermal keratinocytes, cultured to reconstitute a multilayered epidermal model histologically mimicking human skin, while it is both metabolically and mitotically active (https://www.mattek.com/products/epiderm/).

**Figure 7:**
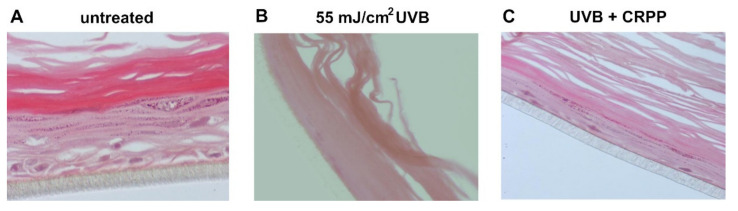
Assessment of the protective effect of CRPP against UVB-induced skin damage. EpidermTM EPI-200 reconstituted skin tissues were treated in the apical surface with 0.1% of CRPP (diluted in assay culture medium) for 2 h, washed with PBS and then exposed to 55 mJ/cm^2^ of UVB irradiation. After UVB exposure, the apical surface of the tissues was incubated with CRPP for 2 h and then washed with PBS. After 24 h, the tissues were harvested and sections were taken. Representative figures of eosin and hematoxylin staining of untreated tissues (**A**), UVB-irradiated skin tissues (**B**) and tissues exposed to UVB and CRPP (**C**). Magnification × 400. (Figure 7A,B are shared images with the respective images, Figure 6A in Karapetsas et al., 2019 [43] and Figure 5B in and Karapetsas et al., 2020 [53] under the common funded project “Greece-China Bilateral R&D Cooperation 2013-2015” NSFR grant; Project Nr. 12CHN167).

The reconstituted skin tissue was pre-treated for 2 h at its apical surface with 0.1% v/v CRPP, irradiated with UVB (55 mJ/cm^2^) then further treated for 2 h with CRPP and subsequently allowed to recover. After 24 h the induced skin lesions and histological pathologies were examined through staining with hematoxylin and eosin. As shown in Figure 6 irradiation of skin tissues with UVB resulted in total necrosis of keratinocytes, while UVB exposure in the presence of CRPP caused only moderate damages such as scattered sunburn cells (pyknotic nuclei) and reducing keratinocytes (Figure 7C).

**Figure 8:**
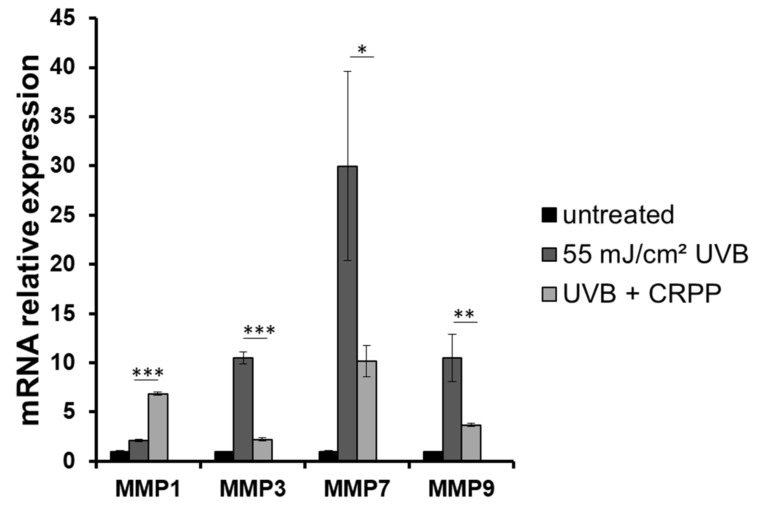
Treatment with CRPP leads to a decrease of the UVB-induced mRNA levels of metalloproteinases (MMPs) in a reconstituted skin model. EpidermTM EPI-200 skin tissues were pretreated with 0.1% CRPP, UVB irradiated (55 mJ/cm^2^) and again incubated with CRPP for 2 h. After 24 h, the tissues were harvested and total RNA was extracted. For the quantification of MMP-1, MMP-3, MMP-7 and MMP-9 mRNA levels in the untreated, the UVB-irradiated and the UVB-CRPP treated skin tissues, real-time PCR was performed. The expression levels of MMP-1, MMP-3, MMP-7 and MMP9 were normalized to those of b-actin. Untreated cells served as reference sample. Each reaction was performed in triplicates. For the relative quantification the formula RQ = 2^-ΔΔCt^ was used. Representative graphs of two independent experiments. * *p* ≤ 0.05, ** *p* ≤ 0.01, *** *p* ≤ 0.001 significantly different from the UVB-irradiated cells.

Next, we examined the potential anti-photoaging properties of CRPP in the reconstitute skin tissue by investigating the expression profile of several MMPs, a group of proteins playing crucial role in the development of photoaging [54]. Thus, through comparative, quantitative PCR, we analyzed the expression levels of MMP-1, MMP-3, MMP-7 and MMP-9 in the irradiated skin tissue samples. Our experiments revealed that the gene expression levels of all the MMPs tested was found to be strongly up-regulated as a result of UVB exposure, with the exception of MMP-1 which exhibited only moderate up-regulation in the irradiated samples. Interestingly, treatment of skin tissues with CRPP significantly decreased the UVB-induced overexpression of the MMPS in all the examined genes except from MMP-1 in which no protection effect from the CRPP was documented (Figure 8).

**Figure 9:**
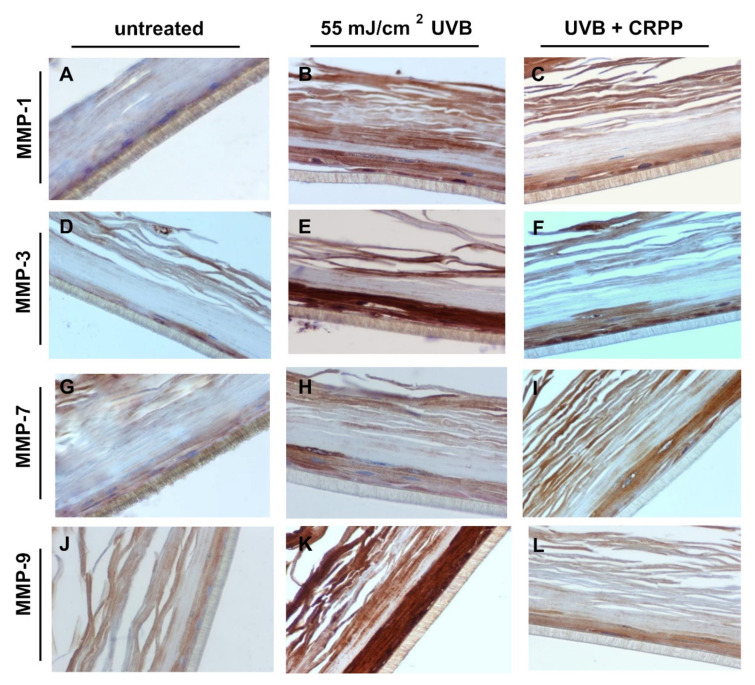
CRPP causes decrease of UVB-induced upregulation of MMPs in a reconstituted skin model. EpidermTM EPI-200 skin tissues were pretreated with 0.1% CRPP for 2 h, UVB irradiated (55 mJ/cm^2^) and again incubated with CRPP for 2 h. After 24 h, the tissues were harvested, sections were taken and immunostaining was performed to detect UVB-induced MMP-1 (**A**–**C**), MMP-3 (**D**–**F**), MMP-7 (**G**–**I**) and MMP-9 (**J**–**L**) positive cells. Representative figures at 400× magnification of untreated skin tissues (A,D,G,J), UVB-irradiated skin (B,E,H,K) and UVB and CRPP treated tissues (C,F,I,L). (Subfigures 9B,D,H,K are shared images with the respective images 8B,E,J,N in Karapetsas et al., 2019 [43]. Subfigures 9A,E,G,J are shared images with the respective images 7A,F,I,M in Karapetsas et al., 2020 [53]. Both studies including the current one, were under the common funded project “Greece-China Bilateral R&D Cooperation 2013–2015” NSFR grant; Project Nr. 12CHN167).

To further validate the anti-photoaging properties of CRPP ex vivo, we also investigated the MMPs expression profile of the UVB exposed skin tissue samples at protein level, via immunohistochemistry. As shown in Figure 9 all of the MMPs which were examined, appeared to be overexpressed in the UVB exposed tissues compared to the non-irradiated control samples, while MMP-3 and MMP-9 exhibited the highest and MMP-7 the lowest up-regulation (Figure 9B,E,H,K). In accordance with the data from quantitative PCR, skin tissues treated with CRPP exhibited significant inhibition of the UVB-induced overexpression of MMP-3 and MMP-9 (Figure 9F,L). On the contrary treatment of skin tissues with CRPP did not led to a significant alteration of MMP-1 and MMP-7 protein levels in comparison to the irradiated non-treated skin tissues (Figure 9C,I).

**Figure 10:**
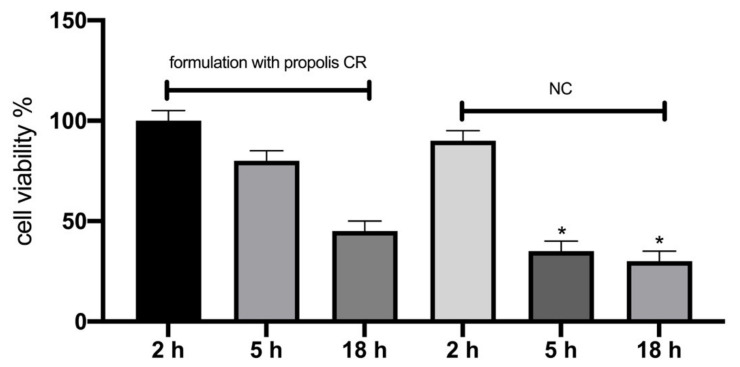
Cell viability levels expressed as mean ± SEM based on reconstituted skin model for: Reconstituted skin model treated with a cosmetic formulation with 1% CRPP and 1% triton-100 as negative control (NC). *—*p* < 0.05 significantly different from the formulation.

Lastly, we evaluated the potential skin irritation of a cosmetic formulation based on CRPP. The cosmetic formulation was an oil in water basic cream base with 1% CRPP incorporated (Figure 10). The parameters measured in vitro were the percentage cell viability using an MTT ET50 assay after topical application of the products for different exposure times (2 h, 5 h and 18 h) according to manufacturer’s instructions. For negative control DPBS was used as well as for positive control 5% SDS was used. For negative control, 1.0% Triton X-100 was used.

## 4. Discussion

The standardization and consistency of metabolite content of natural products is a key issue concerning their bioactivity. Bee products and especially propolis demonstrate high inconsistency because of their dependance on various parameters, such as the flora of the foraging region of the bees, the collection season and the overall health of bee organisms.

We have recently demonstrated the anti-oxidative, anti-mutagenic and anti-ageing properties of propolis extracts, foraged from different parts of Greece, on skin models. In particular, methanolic propolis extracts, exhibited significant antioxidant capacity, as tested by (2,2’-azino-bis(3-ethylbenzothiazoline-6-sulfonic acid))(ABTS) and DPPH in vitro assays. Furthermore, they could protect human keratinocytes from UVB-induced DNA double-strand breaks and decrease the total protein carbonyl content. Interestingly, treatment of a reconstituted skin tissue model with these propolis extracts could prevent the UV-induced upregulation of MMPs at both mRNA and protein level and ultimately alleviate from cellular damages [43]. Thus, propolis and its components are ideal candidates for developing encapsulation and delivery systems with protective and health promoting properties that could be safely applied to human skin.

In the present study we developed a system consisting of liposomes and cyclodextrins incorporating propolis polyphenols, mainly for skin applications. The physicochemical characteristics of the system remain stable over time and when it is stored at a temperature of 6 °C. Furthermore, the antioxidant activity and total phenolic compounds remained stable. The cumulative release of total phenolic compounds was 23.99 ± 1.18% at 8 h and 26.04 ± 2.54% at 48 h. The antioxidant activity, as assessed by DPPH assay, follows this pattern too.

These assays indicate that the system is a stable form of encapsulating and protecting propolis polyphenols. The release rate of the polyphenols that the system demonstrated is suitable for applications where a prolonged provision of polyphenols is desired, such as antioxidant and antiaging targeting.

Next, we examined the anti-mutagenic and anti-oxidative potential of CRPP on a human immortalized skin cell line by employing single cell electrophoresis and measuring the protein carbonyl levels upon UV irradiation and application of the CRPP. Our results demonstrate that CRPP can readily protect HaCaT cells against the mutagenic effects of UV radiation and lower the UV-induced protein carbonyl content, respectively. We further investigated the photoprotective and anti-ageing role of CRPP in a reconstituted human skin tissue model. Application of the CRPP protected the reconstituted skin tissues from UVB-induced lesions and decreased the UVB-induced expression of matrix metalloproteinases, a molecular marker of photoaging, at both mRNA and protein level. 

ATP intracellular levels in NHDF cells in vitro were increased by CRPP under or no UVB irradiation, indicating that CRPP enhanced cell viability, cell proliferation and energy metabolism of dermal fibroblasts. According to previous reports, increased intracellular ATP is associated with higher levels of mitochondrial activity, energy metabolism and cell proliferation and is also indicative of a lack of cytotoxicity [55,56]. Furthermore, in order to explore the gene candidates of biological process related to the addition of CRPP in NHDF cells, we proceed on a differential gene transcription analysis. Specifically, transcripts analysis revealed that the presence of CRPP modified the expression of genes that are associated with cell proliferation, antiaging and antimicrobial response. In our study, we observed that transcripts of TNFα were up-regulated in in NHDF cells treated with CRPP in contrast to control cells. TNF-α is a pleiotropic cytokine with diverse cellular responses [57,58]. Recently, it has been reported that increase expression levels of TNF-α were associated with an antiaging effect on dermal fibroblasts by mediating in ERK/AP--1 signaling pathway [59]. This outcome indicates that CRPP may serve a critical role in the attenuation of skin aging.

Transcripts of Vascular endothelial growth factor (VEGF) were found to be significantly up-regulated in NHDF cells treated with CRPP in contrast to control cells. VEGF is a angiogenesis as well as vasculogenesis related key growth factor indicating that it might also be involved in the regulation of angiogenesis during wound healing [60,61]. These results which are in agreement with a previous study [62], are indicative of a possible positive effect of the CRPP in tissue healing processes, as it is in accordance with previous reports demonstrating that the induction of VEGFA expression correlates with wound healing process [63,64].

Moreover, transcript of AQP3 were upregulated in NHDF cells treated with CRPP in contrast to control cells. This outcome potentially underlines the stimulatory role of CRPP in cell proliferation in an AQP3-dependent manner. It has been reported that transcripts of AQP3 promote cell proliferation related to cell migration process [65,66]. Specifically, Cao C et al. [66]. demonstrated that up-regulation of AQP3 plays an important role in cell proliferation related to wound healing process through facilitating of fibroblasts migration by EGF/EGFR signaling.

IL-4, a multifunctional pleiotropic cytokine mediating in different cellular pathways such as cell proliferation, inflammation, apoptosis [67]. In our study we observed that the transcript levels of il4 are upregulated in NHDF treated with CRPP compared to control. According to a previous report, the increased levels of transcripts of IL4 correlates with the activation of the defense pathways against pathogenic microbes on skin fibroblasts emphasizing the protective role of CRPP against pathogenic microbes of skin [68]. Finally, we observed that the transcript of ITGB2 were upregulated in NHDF treated with CRPP compared to control which potentially demonstrates the in vitro immune protective role of CRPP in human fibroblasts, as increased levels of transcripts of ITGB2 were correlated with immune response [69,70].

Additionally, the reconstituted human skin model was utilized to further confirm the protective properties of CRPP on human epidermis. In our study, the incorporation of CRPP in a cosmetic formulation was proven non-irritant for the epidermis which is an essential step during the development of new skin care related products.

## 5. Conclusions

In the present study we developed a system consisting of liposomes and cyclodextrins incorporating propolis polyphenols, mainly for skin applications. The advantage of the current formulation is that the encapsulation takes place during extraction, so it is a one-pot process, and that the polyphenols are protected from degradation inside the system. Moreover, the encapsulation efficiency is acceptable provided that the raw propolis conforms to specifications regarding its initial polyphenol content, making the system cost effective for upscale applications. Furthermore, once the system is placed in a suitable environment it releases the polyphenol content in a controlled release rate. This fact makes the system ideal for skin applications that demand extended time of contact between skin cells and its ingredients. Such applications are photoprotection, antimicrobial activity and protection against skin aging.

Our results demonstrate that the encapsulation and delivery system can retain the original anti-mutagenic, anti-oxidative and anti-ageing effects of propolis polyphenols to levels similar and comparable to those of propolis methanolic extracts of similar geographic origin as described above, making the system ideal for applications where non-toxic solvents are required and controlled release of the polyphenol content is desired.

## Figures and Tables

**Table 1:** Gene symbol, gene name and primmer sequence.

Gene Symbol	Gene Name	Primmer F (5’-3’)	Primmer R (5’-3’)
ACTB	beta actin	CTGTCCACCTTCCAGCAGATGT	AGCATTTGCGGTGGACGAT
TNFa	Tumor necrosis factor alpha	GCCCTGGTATGAGCCCATCTAT	ATCCCAAAGTAGACCTGCCCA
AQP3	Aquaporin 3	TGTACCAGCTGATGATCGGCT	GATCTGCTCCTTGTGCTTCACA
VEGFα	vascular endothelial growth factor A	CAGACGTGTAAATGTTCCTGCA	ACGTTCGTTTAACTCAAGCTGC
IL4	Interleukin 4	TTCCTGAAACGGCTCGACA	CGTACTCTGGTTGGCTTCCTTC
ITGB2	Integrin subunit beta 2	TGCATCCAGGAGCAGTCGTTT	GGAAGAACCTGCACGGTCACTA

**Table 2:** Cosmetic formulation based on controlled release delivery system of propolis polyphenols (CRPP).

A/A	INCI (EU)	% (w/w)
1	Aqua	71.97
2	Neopentyl Glycol Diheptanoate	4.08
3	Dicaprylyl Ether	3.50
4	Cetearyl Alcohol	3.13
5	Glycerin	3.00
6	Cocoglycerides	3.00
7	Glyceryl Stearate	2.00
8	Propylene glycol	1.65
9	PEG-100 Stearate	1.20
10	CRPP	1.00
11	PEG-20 Stearate	0.67
12	Phenoxyethanol	0.55
13	Tocopheryl Acetate	0.50
14	Panthenol	0.50
15	Sorbitan Stearate	0.50
16	Caprylic/Capric Triglyceride	0.45
17	Caprylyl Glycol	0.46
18	Vitis vinifera leaf extract	0.35
19	Ethylhexylglycerin	0.30
20	Carbomer	0.30
21	Allantoin	0.20
22	Bisabolol	0.20
23	Sodium Hydroxide	0.20
24	Disodium EDTA	0.15
25	Tocopherol	0.15
	Total	100

**Table 3:** Hydrodynamic diameter, polydispersity index and ζ-potential of the system at day 1 and after 1 month at various temperatures.

System	z-Average Diameter (nm)	SD	PI	SD	ζ-pot (mV)	SD
CRPP day 1	255	36	0.355	0.051	−21	5.0
CRPP 25 °C day 30	299	28	0.401	0.012	−16	2.2
CRPP 6 °C day 30	249	51	0.286	0.036	−21	3.0
CRPP 38 °C day 30	402	44	0.216	0.038	−19	3.7

**Table 4:** Evaluation of stability test of TPC: Total phenolic compounds (±SD) and DPPH: Antioxidant activity (±SD) and density (±SD) of the CRPP extract. Values with the same letter in the same column do not show a statistically significant difference, *p* ≤ 0.001.

Week	Parameters	TPC (μg GA/mL)			DPPH (mM Trolox Equivalent)			Density (g/mL)		
T(0)		100.9	±	0.7	33.67	±	0.16	1.0502	±	0.0006
4th week	RT	88.2 ^b,d,e^	±	1.7	29.11 ^d,e,f^	±	0.34	1.0513	±	0.0004
	6 °C	88.9 ^c,d,e^	±	1.7	29.38 ^a,b,c,d^	±	0.49	1.0506	±	0.0004
	38 °C	80.7 ^h^	±	1.2	30.22 ^a^	±	0.23	1.0514	±	0.0003
8th week	RT	85.2 ^a,d,f,g^	±	1.2	27.69 ^h^	±	0.58	1.0509	±	0.0012
	6 °C	88.9 ^a,b,c^	±	0.34	28.28 ^b,e,g,h^	±	0.67	1.0489	±	0.0013
	38 °C	79.4 ^h^	±	2.2	28.84 ^c,f,g^	±	0.79	1.0513	±	0.0012
24th week	RT	70.9	±	0.7	22.13	±	0.72	1.0505	±	0.0005
	6 °C	84.5 ^g^	±	3.9	23.90	±	0.23	1.0497	±	0.0012
	38 °C	61.6	±	0.35	25.62	±	0.47	1.0520	±	0.0013

**Table 5:** Evaluation of stability test of organoleptic characteristics, aspect, color, odor, pH value and refractive index of the CRPP extract.

Week	Parameters	Aspect	Color	Odor	pH (25 °C)	Refractive Index (Brix %)
T(0)		Liquid	Dark Yellow Turbid	Characteristic	6.08	37.38
1st Week	RT	N	N	N	5.99	37.29
	6 °C	N	N	N	6.01	37.01
	38 °C	N	N	N	5.96	37.35
2nd Week	RT	N	N	N	5.66	37.71
	6 °C	N	N	N	6.06	37.77
	38 °C	N	N	N	5.66	38.10
3rd Week	RT	N	N	N	5.88	38.00
	6 °C	N	N	N	6.09	37.58
	38 °C	N	N	N	5.79	37.81
4th Week	RT	N	N	N	5.97	37.53
	6 °C	N	N	N	6.08	37.58
	38 °C	N	M	N	5.80	37.69
8th Week	RT	N	N	N	5.80	38.99
	6 °C	N	N	N	6.05	37.50
	38 °C	N	M	N	5.70	38.01
12th Week	RT	N	N	N	5.72	37.51
	6 °C	N	N	N	5.90	37.53
	38 °C	N	M	N	5.55	37.85
24th Week	RT	N	N	N	5.67	38.08
	6 °C	N	N	N	5.90	37.97
	38 °C	N	M	N	5.56	47.02

Parameters: Different conditions; RT: Room temperature, 6 °C: 6 °C temperature and 38 °C: 38 °C temperature and organoleptic characteristics; color: N—normal/M—modified/IM—intensely modified, odor: N—normal/M—modified/IM—intensely modified and aspect: N—normal/M—modified/IM—intensely modified.

## Data Availability

The data presented in this study are available on request from the corresponding author. The data are not publicly available due to privacy reasons.
